# Examining the Asian American leadership gap and inclusion issues with federal employee data: Recommendations for inclusive workforce analytic practices

**DOI:** 10.3389/frma.2022.958750

**Published:** 2022-09-29

**Authors:** Caroline Goon, Tamara A. Bruce, Janetta Lun, Gabriel Y. Lai, Serena Chu, Phuong-Tu Le

**Affiliations:** ^1^Office of Equity, Diversity, and Inclusion, Office of the Director, The National Institutes of Health, Bethesda, MD, United States; ^2^Division of Cancer Control and Population Sciences, National Cancer Institute, The National Institutes of Health, Bethesda, MD, United States; ^3^Division of Extramural Activities, National Institute of Mental Health, The National Institutes of Health, Bethesda, MD, United States; ^4^Division of Integrative Biological and Behavioral Sciences, National Institute on Minority Health and Health Disparities, The National Institutes of Health, Bethesda, MD, United States

**Keywords:** leadership gap, diversity and equity, workforce analysis, Asian American, workplace inclusion, anti-Asian, National Institutes of Health (NIH)

## Abstract

In April 2021, a coalition of employee resource groups called the Federation of Asian American, Native Hawaiian, and Pacific Islander Network, or FAN, was established at the National Institutes of Health (NIH). The coalition aims to be a unifying voice that represents and serves these diverse communities. Discussion within the group centered around the persistent inequities and the lack of inclusion that the Asian American communities have long endured. Two common themes emerged from these discussions: (1) a leadership gap for Asian Americans in senior leadership and managerial positions, and (2) the everyday experience of exclusion. Asian Americans represent nearly 20% of the NIH permanent workforce yet make up only 6% of the senior leadership positions. These two issues reflect the sentiment that Asian Americans often feel invisible or forgotten in the discourse of structural racism and organizational inequities, especially in organizations in which they are numerically overrepresented. The purpose of this manuscript is to raise awareness of Asian American concerns in the federal workforce and how current employment and workforce analytic practices in this domain might contribute to the invisibility. To accomplish this goal, we will (1) describe relevant historical and contemporary contexts of Asian American experience undergirding their inclusion and visibility concerns; (2) present data analyses from available data sources to provide a deeper understanding of the Asian American leadership gap and lack of inclusion concerns; (3) highlight data availability and analytic challenges that hinder the ability to address the inequity and invisibility issues; and (4) recommend practices in data collection, measurement, and analysis to increase the visibility of this community in the federal workforce.

## Introduction

In 2020, in the wake of the COVID-19 pandemic and the murder of George Perry Floyd, Jr., a new social justice movement began taking hold across the United States (U.S.). The aftermath of the Floyd murder and the ongoing human toll of COVID-19 have brought many persistent issues of racial disparities and inequities to the forefront of the collective consciousness, with many reflecting on the far-reaching systemic barriers faced by marginalized communities due to structural racism (Webb Hooper et al., 2020). During the same period, there was a rise in xenophobic attacks against the Asian American and Pacific Islander communities due to misguided sentiments about the origin of COVID-19. Anti-Asian hate crimes increased 164% between January and March 2021 in comparison to the same period in 2020 (Levin, 2021; Yellow Horse et al., 2021). These attacks challenge the notion that Asian stereotypes and prejudice against Asian communities are relics of the past.

The question of whether Asian Americans are still subject to structural and systemic racism in this country is often juxtaposed with the image of their success. This conundrum is particularly salient in Science, Technology, Engineering, Mathematics, and Medicine (STEMM), as Asian Americans often make up a sizable portion of these workforces. The U.S. federal government, one of the country's largest employers, also has a concentration of Asian American employees in STEMM-related components or agencies. As the National Institutes of Health (NIH) is one such agency, it provides a prime context to explore the discourse and experiences of Asian American government employees which transpired from recent events.

This manuscript draws on a newly established employee resource group (ERG) coalition at the NIH and the ongoing work to highlight policy and data challenges affecting the visibility and accurate understanding of the Asian American experience in the federal government workforce. As part of this ERG coalition, we seek to accomplish four objectives to enhance understanding of the current issues and inspire meaningful actions to elevate equity and visibility for this community: (1) Describe relevant historical and contemporary contexts of Asian American experience undergirding their inclusion and visibility concerns; (2) Present data analyses from available data sources to provide a deeper understanding of the Asian American leadership gap and lack of inclusion concerns; (3) Highlight data availability and analytic challenges that hinder the ability to address the inequity and invisibility issues; and (4) Recommend practices in data collection, measurement, and analysis to increase the visibility of this community in the federal workforce.

## Present definition of the Asian American population

The Asian population in the U.S. is a highly diverse community encompassing over 20 Asian-origin groups (Budiman and Ruiz, 2021). Most of the Asian population in the U.S. belongs to one of six origin groups (Chinese, Indian, Filipino, Vietnamese, Korean, and Japanese). The term Asian American refers to a wide swath of people living in the U.S. who are of an Asian country origin or descendants. We will use the term “Asian American(s)” to reference the highly diverse community of Asians or Asian American populations throughout this article for a few reasons. First, we believe using this term is appropriate for the present purpose because this article focuses on the common, collective experience and treatment of Asians and Asian Americans in the U.S. federal workforce. Acknowledging common experiences does not negate the diversity of ethnicity, cultures, traditions, and experiences within this broad group. We recognize the potential added value of examining Asian subgroups and those who are foreign nationals of Asian countries in the U.S., but examination of this important topic would be outside the current scope of this article. Second, the current race and ethnicity data collection standard of federal employees does not include an option for Asian subgroups to self-identify. Therefore, we have no data to speak to the differences. There has long been a call for disaggregated data for the Asian racial groups in the U.S. Indeed, efforts are being made in various federal demographic data collections to do so. For example, the American Community Survey of the U.S. Census currently includes seven Asian subgroups in their race question^1^ This effort has yet to be fully translated into the practice of personnel data collection.

Another decision made by the authors is that the presented analyses will focus on Asian Americans, and not Native Hawaiians and Pacific Islanders (NHPI). The reasoning is that the NHPI community is a diverse group of indigenous people or descendants of original habitants of Hawaii and other Pacific Islands such as Guam and Samoa that are now U.S. territories. Although many people in this population have Asian ancestry, it would be presumptuous to equate the experiences of the Asian American and NHPI communities similarly, especially in the context of STEMM education and employment. The NHPI data are often suppressed or combined with the Asian racial category due to small numbers and existing policy practices. This is a complex and ever-evolving data issue regarding race and ethnicity in the U.S., which requires more detailed research and analysis in the future. Although this article will not comprehensively represent the NHPI community at the NIH, the overarching goals and mission of the ERG coalition are inclusive of the interests of the very diverse Asian American and NHPI communities at the NIH and we will continue to use this platform to advocate and elevate for the voices of every group.

## National and historical contexts of Asian American experiences

Before we discuss the Asian American workforce at the NIH, it is important to situate this group's experience in the larger national and historical contexts. The Asian American experience of exclusion, including the recent increase in anti-Asian sentiments due to the COVID-19 pandemic, is not a new phenomenon. This section lays out a brief historical and contemporary context of the Asian American experience in the U.S., which undergird the key concerns expressed by the FAN coalition. This is by no means a complete lesson of Asian American history but serves to provide context for the persistent issues that this article focuses on. Specifically, we want to raise awareness that the Asian American experience of exclusion and invisibility is linked to enduring historical and political marginalization. This acknowledgment is important because it is the lens through which current diversity, equity, inclusion, and accessibility (DEIA) policies and practices are formulated.

Over the past few centuries, Asians from various countries of origin have immigrated and settled in the U.S., with records of Filipino Americans first arriving in 1763 (Lee, 2015; Ngai, 2021). In the initial wave, the late nineteenth and early twentieth century saw a rapidly increasing number of Asian immigrants settling in the mainland U.S. or Hawaii—often as indentured servants, laborers, merchants, and with associated family members (Char and Char, 1975; Lee, 2015). They traveled here under various circumstances, and settled in the U.S. for different reasons, with many arriving under the general belief that they would only be in the U.S. temporarily to work and send money back to their families in the homeland. During the same period, national exclusion laws and anti-Asian violence proliferated because many Asian immigrants and Asian Americans were seen to have the quality of “unassimilability” and deemed unfit for the mainstream American culture. The most cited example of this is the Chinese Exclusion Act of 1882, which was the first immigration law that specifically excluded an immigrant group based on race (Lee, 2015; Ngai, 2021). The passage and implementation of such laws and policies, such as the xenophobic Immigration Act of 1924 that excluded entry to anyone born in the continent of Asia, continue to have enduring consequences that impact how Asians are collectively viewed and treated. The internment of Japanese Americans during World War II is another prime example where Japanese Americans, including those who were born and loyal to the U.S., were perceived to be perpetual foreigners and had their loyalty questioned due to the relevant geopolitical conflict.

Changes made in the 1960s to the restrictive immigration laws of years prior ushered in another wave of Asian immigrants from a different set of Asian countries and for different reasons, all of which contributed to the growing diversity of the Asian American mosaic. The legislation allowing exclusion of immigrants from specific national origins was officially struck down, but quotas and preference categories based on family reunification and scarce occupations were still upheld. In 1990, U.S. legislation increased the immigrant cap and expanded on admission categories to include professional visas for high-skilled workers to bolster U.S. overall global competitiveness. Along with the growth in the technology sector, this led to an influx of high-skilled Asian immigrants, many of whom were from India and skilled in science, technology, and engineering. Asian workers in science and engineering occupations increased five-fold from 305,000 in 1995 to 1,543,000 in 2019 (Okrent and Burke, 2021). Voluntary migration with valued skills was not the only source of the increasing Asian American population during this period. The Vietnam War in the 1970s also contributed to the increase of Vietnamese people in the U.S. as they found refuge from the war and subsequent political and economic instability in Vietnam (Harijanto and Batalova, 2021).

Despite the complex and wide array of circumstances of Asian American population growth, the influx of high-skilled workers and strong values in educational attainment among many Asian families yielded a portrayal of success found by Asian Americans. Some Asian American subgroups have a high representation in educational attainment and training pathways for high income earning industries and occupations such as law, business, and STEMM. For example, Asian Americans comprised 10.3% of graduates from the top 30 law schools and are the largest minority group working in major law firms (Chung et al., 2017). Asian Americans also received 9.7% of doctoral degrees in science and engineering in 2018 (National Center for Science Engineering Statistics, 2021), and represented 9.3% of the relevant workforce in 2019 (Okrent and Burke, 2021). Asian Americans represented 21 to 23% of U.S. medical school matriculants and graduates between 2018 and 2021 (Association of American Medical Colleges, 2021) and made up 17.1% of the active U.S. physician workforce in 2018 (Association of American Medical Colleges, 2019). The broad strokes of educational and skilled occupational success among Asian Americans continue to fuel the model minority stereotype, a generalization that Asian Americans are faring well and have successfully overcome systemic racism.

However, this popularized view of Asian American success is not accurate for some segments of the population (Kochhar and Cilluffo, 2018). There are, in fact, many inequalities and variabilities across different Asian American subgroups. For example, in 2015, 72% of Indian American adults obtained at least a bachelor's degree, but only 9% of the Bhutanese did so. The median household incomes of Burmese Americans are half of that of overall Asian Americans (Budiman and Ruiz, 2021). Although the following sections focus on the general representation of Asian Americans in the STEMM workforce, it is important to be cognizant that there are subsets of the Asian American population that remain underrepresented in STEMM.

The national and historical context of the Asian American experience lays out two critical premises for the following discussion about the Asian American workplace experience and workforce analysis. First, Asian Americans as a broad racial group are overrepresented in the STEMM workforce compared to their overall population representation in the U.S., including the workforce at a biomedical research agency like the NIH. However, this broad characterization undermines other disparities that Asian Americans experience, such as representation at leadership levels. Second, Asian Americans are not immune to the effects of racial prejudice and stereotyping. What was once believed to be “unassimilable” and foreign about the Asian race continues to linger in the collective subconsciousness of society, like those beliefs about other racial groups (Banaji and Greenwald, 2016; Payne et al., 2019). Stereotypic views toward Asians, such as the perpetual foreigner syndrome and characterization of Asians as being low in sociability are still very much present today (Wu, 2002; Lin et al., 2005; Sue et al., 2007; Armenta et al., 2013; Yu, 2020).

The anti-Asian hate brought about by the COVID-19 pandemic has reminded us how much the perpetual foreigner stereotype is still alive. It was at this critical juncture that leaders from over 10 ERGs, affinity groups, and scientific interest groups with a focus on the Asian American, Native Hawaiian, and Pacific Islander populations came together and formed a coalition at the NIH. Known as the Federation of Asian American, Native Hawaiian, and Pacific Islander Network^2^, or FAN, the coalition seeks to cultivate an inclusive workplace where Asian American, Native Hawaiian, and Pacific Islander employees at the NIH feel seen, heard, valued, and have equal opportunities to thrive. The coalition also serves as a unifying voice to engage with NIH decision-makers and partner with the broader community to promote DEIA in the NIH workforce. The FAN coalition aims to use data-driven, community-informed approaches to raise awareness and address issues impacting Asian American, Native Hawaiian, and Pacific Islander employees.

## Asian American workforce at the NIH

The NIH is a part of the U.S. Department of Health and Human Services (HHS), supported by close to 19,000 federal employees in its workforce who conduct and support biomedical, behavioral, and health-related research. The agency is comprised of 27 Institutes and Centers, each with its own mission and research agenda, often focusing on specific diseases or body systems. Approximately 80% of the annual NIH budget is used to support research through grants, contracts, and awards to entities outside of the NIH (referred to as Extramural programs). The remaining 20% supports internal research programs and operations (referred to as Intramural programs or Intramural Research Programs [IRP]; Sekar, 2021). The IRP supports ~3,700 scientific investigators and scientists across NIH. The IRP also supports training new generation scientists and hosts about 2,000 trainees during any given fiscal year. These trainees, like federal contractors, are not permanent employees at the NIH and, therefore, they are not included in the federal workforce data and analysis.

Asian Americans have a relatively large presence at the NIH compared with other U.S. federal government agencies. According to the workforce demographic data provided by the NIH Office of Equity, Diversity, and Inclusion (NIH EDI)^3^, Asians/Asian Americans represent 19.8% of the NIH permanent employee workforce in FY2021. Asian/Asian American representation in the NIH workforce is three times higher than their representation in the overall U.S. civilian federal government workforce (19.8 *vs*. 6.4%, FedScope, September 2021). The higher Asian/Asian American representation at NIH is not surprising given the heavy focus on STEMM and medical fields in NIH's mission and operations.

### Asian American workforce analysis at NIH

The establishment of FAN generated robust discussions around the persistent inequities and lack of workplace inclusion issues that the Asian American community has long endured. Two common themes emerged from these discussions. First, compared with other racial and ethnic minority groups, Asian Americans appeared to have more obstacles in reaching senior leadership, supervisory, and managerial roles, which has resulted in an Asian American leadership gap at the organizational level. Second, Asian Americans reported daily encounters that contribute to consistent feelings of being overlooked, ignored, and excluded. We will refer to this latter set of concepts as the everyday experience of exclusion. To find relevant data and analysis to help inform these experiences, the FAN coalition sought out available reports of Asian American workforce data and analysis at the NIH and worked with the NIH EDI office for a more in-depth analysis about the status of Asian American employees.

Our analysis attempted to examine the following questions: What is the status of Asian American leadership gap at the NIH? Furthermore, if a leadership gap exists, does it differ across leadership types (i.e., executive *vs*. managerial), occupations, and organizational units? What do relevant climate surveys and personal accounts say about the workplace experiences of Asian American employees? Does their workplace perception reflect a sense of exclusion or a lack of belonging? While examining these issues with the focus on Asian Americans, we also present and provide the summaries for other racial and ethnic groups. The primary intention is not to compare and suggest one specific group fares worse or better relative to another but to provide transparency and clarity on the statuses of various groups.

### Data sources

The analyses presented below drew from three data sources. The first data source is from the aggregated FY2021 workforce data made available by the NIH EDI office, which includes racial and ethnic demographic data and other major workforce characteristics such as leadership positions, occupations, and organizational units retrieved from the Enterprise Human Capital Management system *via* the NIH internal business intelligence management system called nVision. The collected race and ethnicity data is based on self-identified racial categories provided on the U.S. Office of Personnel Management's Ethnicity and Race Identification form (SF-181 Form) completed by employees during onboarding at NIH or other components of HHS.

The second data source is the NIH IRP Sourcebook that publishes the annual demographic report of NIH employees that have an intramural professional designation^4^ This report presents the demographics of a subset of NIH employees who hold an appointment that, as part of the intramural professional designation, conducts basic and clinical scientific research. Nearly all these employees are recruited through an appointment authority (i.e., Title 42) that are not limited to U.S. citizens or residents. The appointment mechanism allows NIH the flexibility to attract and retain scientists with specialized scientific, technical, and clinical skills. The Title 42 recruitment and hiring process is different from the commonly used appointment mechanism under the General Schedule and Wage Grade pay plans. We used the FY2020 report to inform the current analysis because it was the most recent year of data that provided leadership data (i.e., branch chief).

The third data source is the NIH Federal Employee Viewpoint Survey (NIH FEVS). FEVS is an annual organizational climate survey administered by the Office of Personnel Management (OPM) that assesses federal employees' work experiences and perceptions of their agency's policies, practices, procedures, and leadership. The questionnaire included questions about employees' perceptions and interactions with their agency leadership, their supervisors, and co-workers, as well as their views about the agency's employment policies and programs. We were particularly interested in the New Inclusion Quotient index (New IQ) and its five subindices (i.e., fairness, openness, cooperativeness, support, empowerment). The New IQ consists of 20 items related to inclusive environments (Office of Personnel Management, 2021). Due to the COVID-19 pandemic, many of the items once included in the survey were removed in 2020–2021. Therefore, we examined only the 2019 FEVS data. Due to confidentiality and disclosure risks, the FEVS public database does not provide disaggregated race and ethnicity data at the respondent level, which limits the type of analyses we could use to determine statistical differences. The presented analysis is based on aggregated statistics retrieved from the FEVS Analysis on Demand system, which could still be useful for observing general patterns. In addition to the FEVS data, we also draw on the narratives and stories collected from the diverse FAN communities to provide relevant qualitative evidence of their experiences.

For the following data presentation, we will use the term “Asian/Asian American” to present the Asian American data. This is because the workforce and survey data analyses presented below was based on data collection of the self-identified racial category of “Asian” and not specifically Asian Americans. For example, OPM's SF-181 Form defines the racial category of Asian as “a person having origins in any of the original peoples of the Far East, Southeast Asia, or the Indian subcontinent including for example, Cambodia, China, Indian, Japan, Korea, Malaysia, Pakistan, the Philippine Islands, Thailand, and Vietnam.” We want to be accurate in our data presentation and respectful to our fellow colleagues who self-identify as Asian but not Asian American.

### Lacking Asian American representation in managerial and executive leadership

Despite the large representation of Asians/Asian Americans in the NIH workforce, Asian/Asian American representation in managerial or executive leadership positions was observed to be notably lower compared to the overall workforce. Asians/Asian Americans made up only about 6% of the senior leadership positions while representing 21.2% of those in non-leadership positions in fiscal year 2021 (see Table 1). We defined managerial and executive leadership positions as those federal positions that have structural and managerial authorities. In the workforce data, senior leaders are individuals who have the authorities to make organizational, management, and policy decisions, which include hiring, setting strategic priorities, policy and program decisions, and resource allocations. All of these could affect employment opportunities, operational procedures and practices, workplace climate, and reward structures. To examine a broader set of leadership positions at the NIH, we also examined the supervisor and manager workforce that includes employees who have a supervisory status. Similar to what we found with senior leaders, Asians/Asian Americans represented 13.7% of the supervisory workforce, which was half of their representation among non-leadership staff of 21.2% (see Table 1). Using a *z*-test comparison, the proportional difference was statistically significant at α = 0.01 level with Bonferroni adjustment for multiple comparisons (Bland and Altman, 1995), *z* = 9.78, *p* < 0.001.

**Table 1:** Leadership gaps of employees by race and ethnicity in fiscal year 2021.

	**Non-leadership positions benchmark**		**General leadership positions**			**Senior leadership positions**		
	***N***	**%**	***N***	**%**	**% Point diff from Non-leader**	***N***	**%**	**% Point diff from Non-leader**
Non-Hispanic or Latino/a								
Asian or Asian American	3239	21.2%	451	13.7%	−7.5%†	9	6.0%	−15.2%†
Black or African American	3391	22.2%	432	13.1%	−9.1%†	14	9.4%	−12.8%†
White	7724	50.5%	2251	68.4%	17.9%†	121	81.2%	30.7%†
All other races	301	2.0%	41	1.2%	−0.7%	^*^	^*^	^*^
Hispanic or Latino/a	625	4.1%	114	3.5%	−0.6%	5	3.4%	−0.7%

Non-leadership, general leadership, and senior leadership samples are distinct, and combine to make up the total workforce. Leadership positions are defined as follows for each group type: Senior leadership includes titles of NIH Director, Deputy Director, and Associate Director, and IC Director, Deputy Director, Executive Officer, Clinical Director, and Scientific Director, and aligns with HR data on Top five positions with the exclusion of Scientific Executives; General Leadership includes employees whose positions are coded under Supervisory Status Codes 2 (Supervisor or Manager), 4 (Supervisor), and 5 (Management Official), as defined by OPM regulation and not included in Senior Leadership roles; Non-Leadership includes all other employees not included in General or Senior Leadership. Data for employees represented in this reporting are self-identified; those classified in the five racial groups and two or more race group are all non-Hispanic or Latino/a. Hispanic or Latino/a employees are included in that category regardless of their race selection(s). To maintain confidentiality and protect individual identification from deductive disclosure risk, values of <4 are suppressed for reporting purposes and designated with an asterisk. Data retrieved from NIH Office of Equity, Diversity, and Inclusion for FY2021. Additional information about these data located at https://www.edi.nih.gov/data/demographics. The dagger symbol (†) indicates the proportion of the leadership positions was significantly different from the proportion of the non-leadership positions at the α = 0.01 level.

The lack of representational diversity at the leadership level is not a unique experience to Asian American employees. As Table 1 shows, Black/African Americans' leadership representation was also lacking to a similar extent to that of Asian/Asian Americans, *z* = 11.71, *p* < 0.001. However, Hispanic representation at the executive and managerial positions were nearly on par with their non-leadership staff representation, *z* = 2.13, *p* = 0.03. Overall, non-Hispanic Whites were the only racial group having a greater representation at the senior and general leadership levels than their representation at the staff level, *z* = −18.68, *p* < 0.001, and *z* = −7.46, *p* < 0.001, respectively.

#### Asian American leadership gap by occupation types

Next, we examined whether the Asian American leadership gap varies by the type of work employees engage in, defined by their occupation type and leadership level. Specifically, we investigated Asian American representation among supervisory and manager roles across the three occupational types (i.e., scientific, health professional and technical, and administrative and management) and compared those to their representation at the non-leadership level.

Scientific occupations, as mentioned before, include positions that directly lead or conduct basic or clinical research or provide scientific oversight for externally funded research. The most populous occupational series that falls under this category are the 0401 General Natural Resources Management and Biological Sciences series and 0601 General Medical and Healthcare. Health professional and technical occupations include positions of allied health professions, such as nurses, pharmacists, biological lab technicians, and patient care technicians. Administrative and management occupations include positions that provide management and infrastructural-related support to the agency operations.

Asians/Asian Americans represented 32.6% of the scientific occupations at the non-leadership or staff level, but only 17.5% of the general leadership level (a difference of 15.1%), *z* = 12.77. *p* < 0.001, which is nearly a two-fold disparity. Compared to the difference in the scientific occupations, the difference was smaller in the health professional and technical occupations as well as the administrative and management occupations (a difference of 5.7 and 2.2%, respectively). These differences were not statistically significant, *z* = 1.76, *p* = 0.08 and *z* = 2.36, *p* = 0.02. Black/African American employees also showed a leadership gap but the gap pattern across occupation types differed from that of Asians/Asian Americans (see Table 2), *z'*s > 2.99, *p*'s < 0. 001. Once again, non-Hispanic White was the only racial and ethnic group having a greater representation at the general leadership level than at the staff level, *z*'s < −0.4.81, *p*'s < 0. 001.

**Table 2:** Leadership gaps by race and ethnicity by occupation type in fiscal year 2021.

	**Non-leadership positions**						**General leadership positions**								
	**Scientific**		**Health professional and technical**		**Administrative and management**		**Scientific**			**Health professional and technical**			**Administrative and management**		
	***N***	**%**	***N***	**%**	***N***	**%**	***N***	**%**	**% Point diff from Non-leader**	***N***	**%**	**% Point diff from Non-leader**	***N***	**%**	**% Point diff from Non-leader**
Non-Hispanic or Latino/a															
Asian or Asian American	2236	32.6%	325	16.5%	678	10.5%	331	17.5%	−15.1%†	15	10.8%	−5.7%	105	8.3%	−2.2%
Black or African American	465	6.8%	626	31.9%	2300	35.6%	93	4.9%	−1.9%†	25	18.0%	−13.9%†	314	24.9%	−10.7%†
White	3820	55.7%	913	46.5%	2991	46.3%	1389	73.5%	17.8%†	94	67.6%	21.2%†	768	61.0%	14.6%†
All other races	83	1.2%	38	1.9%	180	2.8%	14	0.7%	−0.5%	*	*	*	25	2.0%	−0.8%
Hispanic or Latino/a	256	3.7%	63	3.2%	306	4.7%	63	3.3%	−0.4%	*	*	*	48	3.8%	−0.9%

Non-leadership and leadership samples are distinct and combine to make up the total workforce excluding Senior Leadership. General Leadership includes employees whose positions are coded under Supervisory Status Codes 2 (Supervisor or Manager), 4 (Supervisor), and 5 (Management Official), as defined by OPM regulation and not designated with Senior Leadership roles; non-leadership includes all other employees not included in General or Senior Leadership. Scientific occupations directly lead or conduct basic or clinical research or provide scientific oversight for externally funded research; Health professional and technical occupations include positions of allied health professions, such as nurses, pharmacists, biological lab technicians, and patient care technicians. Administrative and management occupations include positions that provide management and infrastructural-related support to the agency operations. Percentages calculated as proportion of Asians/Americans with detailed criteria out of all employees with such criteria (e.g., The 2,236 Asian/Asian American employees represented 32.6% of the 6,860 scientific employees in non-leadership positions). Data for employees represented in this reporting are self-identified; those classified in the five racial groups and two or more race group are all non-Hispanic or Latino/a. Hispanic or Latino/a employees are included in that category regardless of their race selection(s). To maintain confidentiality and protect individual identification from deductive disclosure risk, values of <4 are suppressed for reporting purposes and designated with an asterisk. Data retrieved from NIH Office of Equity, Diversity, and Inclusion for FY2021. Additional information about these data located at https://www.edi.nih.gov/data/demographics. The dagger symbol (†) indicates the proportion of the leadership positions was significantly different from the proportion of the non-leadership positions at the α = 0.01 level.

We also examined the Asian American leadership gap in a subset of the scientific workforce that are specialized scientists and clinicians who directly lead and conduct basic and clinical research. Using the personnel demographic data provided by the NIH IRP, we examined the proportion of Asian/Asian Americans who were principal investigators and branch chiefs. This included those who were Asian foreign nationals. Principal investigators often operate as independent researchers responsible for a research program and have similar roles to the faculty at an academic or research institution. Branch chiefs are principal investigators who lead the operations of multiple research programs and have similar roles to academic department chairs. In 2020, Asians/Asian Americans represented 19.6% of all the NIH IRP principal investigators (22% excluding those who were also branch chiefs), but only 7.1% of the more senior level branch chiefs, *z* = 4.64, *p* < 0.001, which is nearly a three-fold disparity. Black/African Americans and Hispanics represented 2.5 and 4.5% of the principal investigators respectively, and 2.2 and 6.0% of the branch chiefs, respectively. Proportion comparison tests showed no statistically significant differences for these two racial and ethnic groups, *z* = 0.25, *p* = 0.80 for Black/African Americans, and *z* = −0.90, *p* = 0.37 for Hispanics. Aside from the low representation of Black/African American and Hispanic scientific investigators overall, their representation at the branch chief level were comparable to those at the principal investigators level.

#### Asian American leadership gap in scientific occupations by organizational units

Given that we found a larger Asian American leadership gap among employees in scientific occupations, we wanted to examine if this difference varied by organizational units. Specifically, we considered whether the leadership gap in the scientific workforce at NIH differed for those who work in the extramural units compared to the intramural and other units with cross-cutting functions. Almost 70% of the scientific occupations belong to one of the two major job series: Series 0401 General Natural Resources Management and Biological Sciences, and Series 0601 General Medical and Healthcare. We focused the following analysis on these two major scientific occupations.

As shown in Table 3, proportions of Asians/Asian Americans in leadership levels differed based on organizational unit. In the extramural research units, Asians/Asian Americans represented 26.7% of the non-leadership scientific employees and only 15.2% of those in a supervisory and managerial role, *z* = 4.68, *p* < 0.001. In the intramural research units, Asians/Asian Americans represented 40.3% of the non-leadership scientific employees but only 24.3% of those in a supervisory and managerial role, *z* = 6.62, *p* < 0.001. A noteworthy finding is that the leadership gap was two times greater (−16.0%) in the intramural research units compared with the cross-cutting units (−7.2%) with the difference from extramural research units in between (−11.5%). The difference in the cross-cutting units were not statistically significant, *z* = 2.21, *p* = 0.03. In summary, the representation of Asians/Asian Americans at the supervisory and managerial positions in the two major scientific occupations varied across different organizational units. Compared with other racial and ethnic groups, this gap was more consistent among Asians/Asian Americans across the organizational units.

**Table 3:** Leadership disparities in scientific occupation series 0401 and 0601 by race and ethnicity by organization unit type in fiscal year 2021.

	**Non-leadership positions**						**General leadership positions**								
	**Extramural**		**Intramural**		**Other cross-cutting**		**Extramural**			**Intramural**			**Other cross-cutting**		
	***N***	**%**	***N***	**%**	***N***	**%**	***N***	**%**	**% Point diff from Non-leader**	***N***	**%**	**% Point diff from Non-leader**	***N***	**%**	**% Point diff from Non-leader**
Non-Hispanic or Latino/a															
Asian or Asian American	438	26.7%	1094	40.3%	137	21.3%	57	15.2%	−11.5%†	115	24.3%	−16.0%†	27	14.1%	−7.2%
Black or African American	163	9.9%	121	4.5%	82	12.7%	26	6.9%	−3.0%	20	4.2%	−0.2%	19	9.9%	−2.8%
White	954	58.1%	1379	50.8%	381	59.2%	283	75.3%	17.2%†	314	66.2%	15.5%†	133	69.3%	10.1%
All other races	15	0.9%	27	1.0%	15	2.3%	*	*	*	*	*	*	4	2.1%	−0.2%
Hispanic or Latino/a	72	4.4%	94	3.5%	29	4.5%	8	2.1%	−2.3%	22	4.6%	1.2%	9	4.7%	0.2%

General Leadership includes employees whose positions are coded under Supervisory Status Codes 2 (Supervisor or Manager), 4 (Supervisor), and 5 (Management Official), as defined by OPM regulation, and not designated with Senior Leadership roles; non-Leadership includes all other employees not included in General or Senior Leadership. Organizational unit type is based on the organizational standard administrative code (SAC). The extramural units included offices, divisions or branches that primarily support extramural activities such as funding opportunities development, peer reviews of grants applications, and grants and contracts managements to outside institutions. The intramural units included offices, divisions, branches, and laboratories that directly conduct or support basic and clinical research. The remaining units are referred as other cross-cutting units because many of these units serve extramural and intramural functions at the NIH, which include human resources, information and technology support, infrastructure and facilities maintenance, and other administrative managements. Occupational codes 0401 and 0601 are defined by the OPM occupational handbook. Percentages calculated as proportion of Asians with detailed criteria out of all employees with such criteria (e.g., The 1,094 Asian/Asian American employees represented 40.3% of the 2,715 scientific employees who were in the 0401 or 0601 occupational series and Intramural organizational units). Data for employees represented in this reporting are self-identified; those classified in the five racial groups and two or more race group are all non-Hispanic or Latino/a. Hispanic or Latino/a employees are included in that category regardless of their race selection(s). To maintain confidentiality and protect individual identification from deductive disclosure risk, values of <4 are suppressed for reporting purposes and designated with an asterisk. Data retrieved from the NIH Office of Equity, Diversity, and Inclusion for FY2021. Additional information about these data located at https://www.edi.nih.gov/data/demographics. The dagger symbol (†) indicates the proportion of the leadership positions was significantly different from the proportion of the non-leadership positions at the α = 0.01 level.

### Everyday experience of exclusion and invisibility

The second persistent issue faced by Asian American employees at the NIH, as identified by the FAN coalition, is the everyday experience of exclusion and invisibility. These experiences are often characterized by routine social interactions that convey and reinforce Asian stereotypes and marginalization such as the stereotypes of model minority and perpetual foreigners. During the 2022 NIH Asian American, Native Hawaiian, and Pacific Islander (AA and NHPI) Heritage Month, NIH FAN collected stories and quotes from the AA and NHPI community about what has been misunderstood about them. These stories and quotes were published in an online video on the NIH EDI website^5^ Many of these stories reflect experiences of being seen as an outsider, or a perpetual foreigner, living under expectations of being a high achiever especially in STEMM subjects, and how others seemed to lack understanding about the multitude of cultures contained in the Asian diaspora. Although it was a relatively small sample of anecdotal evidence and was not systematically collected, the Asian experience expressed in these stories of being on the receiving end of subtle or overt forms of racism was remarkably consistent with those documented in social science and psychology literature (Sue et al., 2007; Huynh et al., 2011; Armenta et al., 2013; Nadal et al., 2014). Perhaps what is less known and discussed is how these experiences insidiously shape workplace interactions and equity for Asian American employees.

In search of more evidence of the Asian American workplace experience and perception at the NIH, we turned to the results from the 2019 NIH FEVS. We examined the survey results of the New IQ index and its five subindices (i.e., fairness, openness, cooperativeness, support, empowerment). Table 4 shows the heat map of the average percent agreement for the overall New IQ index and its sub-indices by racial and ethnic groups. The results showed little differences across racial and ethnic groups. All racial groups tended to express lower agreement with fairness and greater agreement with being supported by their supervisors (i.e., supportive). There were some variations across racial groups with respect to fairness. Overall, Asian/Asian American employees at the NIH, according to the FEVS, reported feeling well-respected and included. If anything, they reported feeling equally included as White employees and more so than other employees of people of color.

**Table 4:** Heat map of the average percent agreement for the New Inclusion Quotient (IQ) index and sub-indices from the 2019 NIH Federal Employee Viewpoint Survey by race and ethnicity.

	**New inclusion quotient (IQ)**					
	**Overall**	**Sub-index**				
		**Fair**	**Open**	**Cooperative**	**Supportive**	**Empowering**
Non-Hispanic or Latino/a						
Asian or Asian American	77	69	77	74	85	78
Black or African American	69	57	66	71	81	71
White	74	64	76	71	85	73
All other races	65	54	64	61	80	66
Hispanic or Latino/a	72	63	74	68	84	71

Data for employees represented in this reporting are self-identified; those classified in racial groups are all non-Hispanic or Latino/a. Red color indicates low values (lower agreement) and green indicates high values (greater agreement). Data retrieved from the FEVS Analysis on Demand system at https://www.dataxplorer.com/ for the 2019 administration year. Percent agreement was calculated based on the combined percentages of respondents who answered positively, i.e., Strongly Agree or Agree; Very Satisfied or Satisfied; or Very Good or Good, depending on the item's response categories, divided by the total number of positive, neutral, or negative responses. The number of responses for each racial and ethnic categories are the following: Asian 1,614; Black or African American 1,574; White 5,919; All other races 265; Hispanic or Latino/a 503. Due to protection of confidentiality, the results of Native Hawaiian and Pacific Islander respondents cannot be presented separately.

## Discussion on Asian American (in)visibility in workforce data and analysis

Following the rise of violence against Asian communities, in May 2021, the U.S. federal government issued a new executive order to end anti-Asian hate and increase federal efforts to ensure equity, inclusion, and racial justice for the diverse Asian American, Native Hawaiian, and Pacific Islander communities including those in the federal workforce^6^ The present perspective and data analysis provided by the NIH Federation of Asian American, Native Hawaiian, and Pacific Islander Network (FAN) is aligned with this recent policy directive. The lack of Asian American representation in leadership levels is still present, at least within the NIH workforce. The analysis results were less conclusive regarding the everyday experience of exclusion. Although qualitative inputs were largely consistent with previous literature about those experiences, results based on a governmental employee survey suggest that these experiences were not significantly affecting Asian American employees' views of their workplace culture.

These workplace issues shared by the NIH Asian American community is not unique to the agency described in this paper and have been documented in other industries and sectors. Yet, what is notable is the persistent lack of progress in addressing these workplace issues faced by the community. For instance, the leadership gap experienced by the NIH principal investigators was discussed and reported in 2005 by the late NIH scientist Dr. Kuan-Teh Jeang. At that time, while 21.5% of the 280 tenure-track investigators were Asian/Asian American, only 9.2% of the 950 senior investigators were Asian/Asian American, and only 4.7% of the 200 lab or branch chiefs were Asian/Asian American (Mervis, 2005). In a parallel review of the 2020 data, 22.4% of the 219 tenure-track investigators, 18.9% of the senior investigators, and 7.1% of the 183 branch chiefs were Asian/Asian American. This rough comparison suggests a considerable increase in promotions since 2005 for Asian/Asian Americans at the tenure-track investigator level to the senior investigator level (+9.7%), but that growth was modest at the branch chief level (+2.4%).

Beyond the NIH, in 2008, the Asian American leadership gap in the government was documented by the U.S. Equal Employment Opportunity Commission's (EEOC) Asian American and Pacific Islander Work Group Report. In the EEOC review and analysis, Asian and Pacific Islander participation rates in executive and senior leadership positions hovered at 4.4 and 7.1%, for HHS and the NIH, respectively, while they comprised 7.2 and 13.5% respectively, of the permanent workforce (The U. S. Equal Employment Opportunity Commission, 2009). Previous reports and the present research showed that while Asian American representation in the workforce increased, their participation rate at the leadership level remained relatively the same. Given the greater participation of Asian Americans in the STEMM workforce, it would be worthwhile for future research to examine the leadership gap across STEMM-related government agencies (e.g., Department of Defense, National Aeronautics and Space Administration, Food and Drug Administration, etc.).

From the analysis and findings, we draw attention to the observation that the Asian American leadership gap is not uniform across occupation types and organizational units, which suggests opportunities for further examination of barriers and solutions. This highlights the importance of grounding DEIA workforce analysis in organizational and structural contexts and providing transparency around any relevant barrier analyses that track progress (or lack thereof). For instance, we found that the managerial or general leadership gap among Asian American employees is greater in the scientific occupations and within the intramural program. This observation could lead to further analyses on understanding the root causes of the leadership gap. It is plausible that the gap is related to different promotion practices for various appointment mechanisms across programs (e.g., Title 42 *vs*. Title 5) with some of the practices allowing hidden biases to influence leadership advancement and selection. Another possible cause for this gap is related to differences in cultural expectations and characteristics of leadership. Many Asian Americans may not be aware, or discouraged by, the conflicting cultural ideals and expectations about career progression and leadership to navigate upward mobility and advancement to a leadership position in some occupations (House et al., 2004; Sy et al., 2017). These nuances call for a comprehensive structural and culturally informed approach to understand and address this leadership gap.

**Figure 1:**
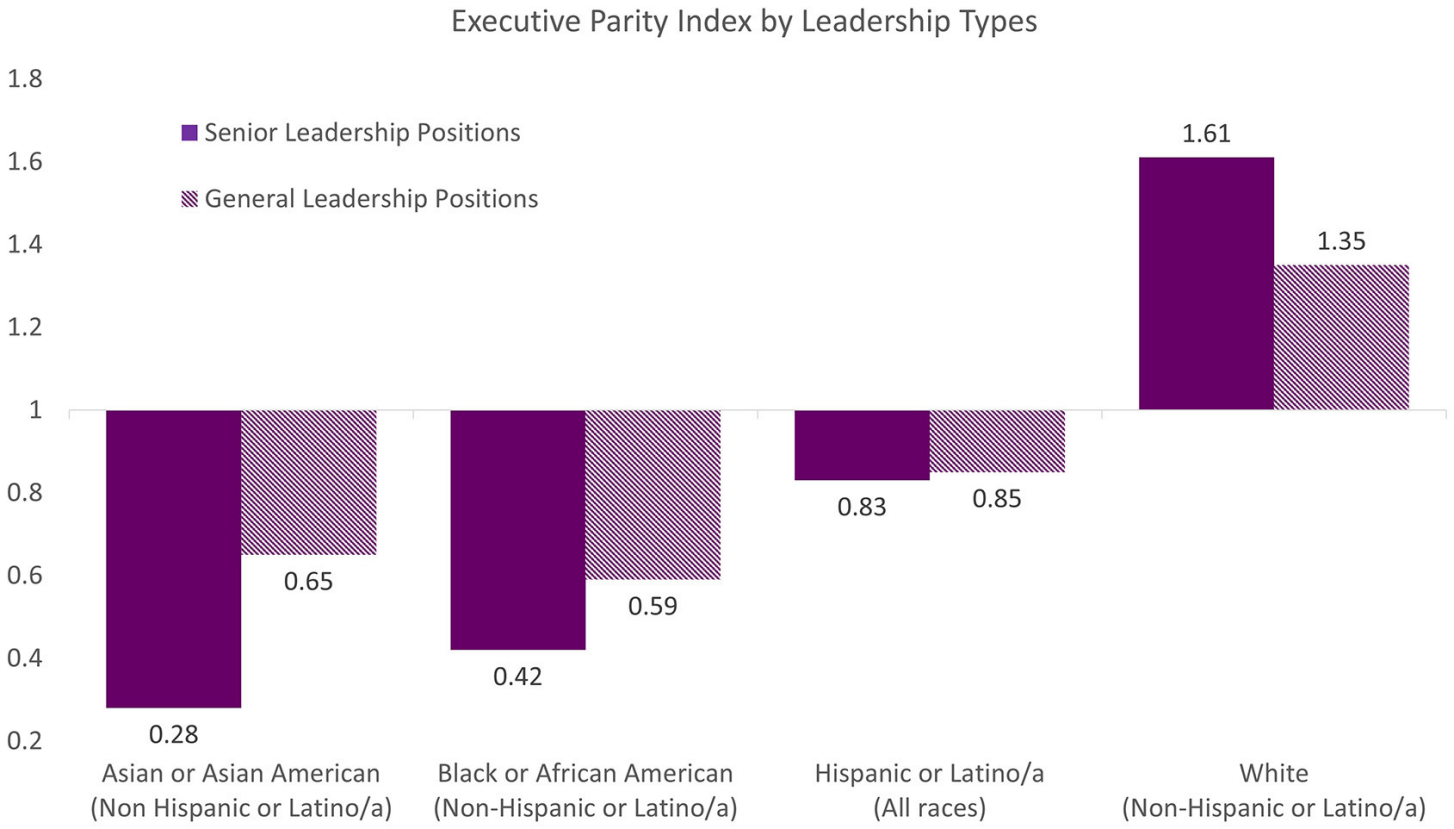
The Executive Parity Index (EPI) is a metric created by Ascend, a non-profit Pan-Asian business professional organization, to illustrate the ratio of one group's representation at the executive level of an organization versus its representation at the relevant professional level. An EPI value of 1.0 indicates that the group's representation at the level of interest is at parity with those of the relevant professional level. An EPI less than 1.0 indicates there is underrepresentation at the level of interest, or lower than expected, in reference to their representation at the relevant pool. In contrast, an EPI greater than 1.0, indicates there is overrepresentation, or more than expected, in reference to their representation in the relevant pool.

To facilitate this type of analysis and reporting, there is a wide assortment of workforce data metrics presented by professional groups and organizations. One example includes the Executive Parity Index (EPI) metric, which was created by Ascend, a non-profit Pan-Asian business professional organization (Gee et al., 2015). The EPI has been used to illustrate the ratio of one group's representation at the level of interest (e.g., executive, senior leadership, directors) *versus* its representation at the relevant professional level. Figure 1 shows an example of applying the index to illustrate the NIH leadership gap data. The utility of metrics such as the EPI is to encourage detection and tracking of under- or over-representation at any given level or group of employees with respect to the relevant professional pool within an organization. This practice not only increases ease for reporting and interpreting data, but it also reinforces the consideration of using relevant benchmarks for intended analyses. As a case in point, the FAN coalition highlighted the fact that larger discussions of workforce representation, from time to time, completely ignored the Asian American leadership gap. This is because Asian Americans are considered overrepresented or sufficiently represented in the workforce with respect to the U.S. population. The lack of grounding within the organizational context has added to the invisibility of Asian Americans in overall DEIA efforts and subsequent implementation plans.

Finally, the invisibility of concerns from the Asian American community can also be attributed to the lack of effective workplace culture and inclusion assessments that could accurately capture Asian Americans' and other groups' workplace experience in the federal workforce. The FEVS data suggests that Asian American employees did not face workplace exclusion. However, this conclusion is tenuous because none of the New IQ questions properly captured the everyday experience of exclusion and invisibility that Asian American employees have expressed qualitatively. Without meaningful assessment tools and instruments that can precisely capture everyday experiences in the workplace, we are limited in our ability to promote a truly inclusive workplace culture and the gaps in understanding where these perceptions of bias will continue to persist unaddressed. Joan Williams and colleagues' work on women leadership gaps and career advancement provides an example of how meaningful assessment tools are helpful. Williams and her colleagues developed the bias-interrupter toolkits that help organizations and managers identify specific behaviors and practices that perpetuate gender inequities (William et al., 2022). The toolkits include survey instruments that assess specific workplace behaviors and experiences such as being interrupted during meetings, having ideas being ignored, or being tasked with office support work. As these assessments inform specific and modifiable behaviors, management can then adjust and drive meaningful changes in workplace culture. A similar research and implementation approach can be adopted to address the everyday experience of exclusion experienced among Asian American employees.

## Recommendations to increase visibility of Asian American experience through inclusive data analytic practices

Historically, the Asian American experience is described heavily through the lens of immigrants and foreigners, even though many of these communities have long lived in and made invaluable contributions in building up this country. Among the many DEIA efforts, including those at NIH, the issues that Asian Americans experience are not often prioritized because they appear to be “well-represented” in the workforce, and/or these issues have been masked by the model minority stereotype because of the broad characterization and lumping of the diverse experiences. Asian Americans may have appeared to be highly satisfied with their jobs and reportedly did not experience any racism or discrimination as shown by the survey data. Yet, the data does not capture workplace biases specific to this very group. Taken together, these issues obscure the true nature of the Asian American experience and help perpetuate the cycle of invisibility (Sue et al., 2021).

As a biomedical research agency, the NIH relies on data to identify problems and solutions. However, if research methodology and practices such as data measurement, reporting and analysis are fundamentally biased and, as a result, continue to reproduce conclusions that perpetuate structural racism, then we as NIH scientists and staff are not empowered to be in a strong position to affect change. Data measurements and analytic practices are cultural products that can be changed and adapted for the better. With consideration from this perspective, we make the following three recommendations toward a more inclusive data analytic practice of federal and DEIA workforce analysis for Asian Americans.

Recommendation #1: Analyze and use relevant metrics to interpret Asian American data consistently.

During the process of searching and compiling data for this manuscript, we encountered examples of data reporting that may have contributed to the discussed barrier of invisibility. Sometimes, workforce data presentations and analyses omit the Asian or Asian American data or combine the Asian American data with other groups (e.g., Asian and Pacific Islander, Asian and White combined as well-represented groups in STEMM). When data has been made available, the focus tends to be on overall representation. As Asian Americans are often deemed overrepresented in the STEMM workforce, additional and more robust barrier analyses are often stopped short. Using relevant metrics such as the EPI described above can be helpful to consistently probe and detect inequities within an organization. Also, there is minimal effort to examine the intersectional experience of Asian Americans, which is a long-standing challenge, especially in terms of accessing and analyzing disaggregated data. Therefore, as a first step, regardless of the overall representation of Asian Americans in the workforce, a standardized practice to analyze and present Asian American workforce data or relevant disaggregated data should be implemented, whenever it is possible and appropriate to do so.

Recommendation #2: Conduct rigorous barrier analyses of the Asian American leadership gap that are grounded in the organizational context.

Recruitment and employment barrier analysis of Asian Americans should 7 receive equitable attention in workforce analyses. Sometimes, the analyses regarding the career trajectory of Asian Americans are not prioritized because they are deemed appropriately or overrepresented in the overall workforce. As shown in the present analysis, the lack of Asian American representation at the senior and general leadership level appears to be concentrated in scientific occupations and the intramural program at the NIH. This finding suggests that there are nuances and intersectional experiences underlying this gap. To better understand the complexity of factors hindering career progression and upward mobility, additional rigorous analyses that consider other organizational and cultural variables are needed. These considerations should include organizational structures, cultural differences in leadership expectations, and/or current events that could have a significant impact on Asian Americans (e.g., COVID-19 pandemic, anti-Asian hate crimes). Such understanding is a critical step toward identifying the causes that underlie the persistent leadership gap and addressing the challenge of invisibility faced by the Asian American community.

Recommendation #3: Improve workplace culture assessments to reflect the Asian American workplace experience accurately.

There needs to be more culturally informed approaches to developing and implementing workplace climate and inclusion assessments. Qualitative data collection to inform measurement development and conducting regular reviews of workplace climate assessments with a cultural lens can be immensely helpful in understanding better the perspectives on how different racial and ethnic groups, such as Asian Americans, perceive climate and inclusion at their workplace. Culturally responsive assessments are essential tools to understand whether and how implicit stereotypes, unspoken expectations, and behavioral norms such as assertive communication styles common in Western cultures could hinder upward mobility for Asian Americans. Establishing and empowering employee resource groups (ERGs) to gather feedback and concerns about ongoing workplace climate issues is another way to realize this recommendation. Effective ERGs can provide a safe space for employees to identify the DEIA needs of their communities while augmenting the organizational leadership efforts to successfully implement program that address those needs.

## Conclusion

Overall, the challenges and recommendations presented in this paper call for a better conceptual and inclusive DEIA assessment framework. A more robust assessment and evaluation framework is needed to propel actions and progress to ensure equity and inclusion in the federal workforce. This manuscript contributes to this discussion by pinpointing gaps in measurement and data analytic practices that could impede an organization's ability to enact meaningful changes to enhance DEIA. Dismantling structural and institutional racism requires us to question the assumptions of our current ways of measuring and assessing group differences and expand these efforts. Who decides on what needs to be measured? What questions need to be asked, and then answered? What is the data telling us? Through this critical lens of our current practices, we can break the vicious cycle of systemic inequities. We believe that the re-thinking of the current DEIA assessment framework and approaches not only benefit the Asian American community, but for all.

## Author contributions

CG, TB, and JL contributed to conception and data interpretation of the review. TB retrieved the data. TB and JL conducted the statistical analyses. CG and JL wrote the first draft of the manuscript. GL, SC, and P-TL wrote sections of the manuscript. All authors contributed to manuscript revision, read, and approved the submitted version.

## Conflict of interest

The authors declare that the research was conducted in the absence of any commercial or financial relationships that could be construed as a potential conflict of interest.

## Footnotes

1 Available online at: https://www.census.gov/acs/www/about/why-we-ask-each-question/race/.

2 Available online at: https://www.edi.nih.gov/blog/news/nih-aa-nhpi-group-moves-forward-momentum-%E2%80%93-and-mission.

3 The NIH workforce presented in this paper includes only civilian federal employees who have an onboard classification of permanent or temporary full-time, and part-time or intermittent. Data were unavailable for contractors, post- and pre- doctoral trainees, post-baccalaureate trainees, students, and volunteers. Available online at: https://www.edi.nih.gov/data/demographics for more information.

4 Available online at: https://oir.nih.gov/sourcebook/personnel.

5 Available online at: https://www.edi.nih.gov/people/sep/aapi/campaigns/aanhpi-heritage-month-2022.

6 Available online at: https://www.whitehouse.gov/briefing-room/presidential-actions/2021/05/28/executive-order-on-advancing-equity-justice-and-opportunity-for-asian-americans-native-hawaiians-and-pacific-islanders/.
